# Are crystal structures getting better? A temporal and elemental analysis of the Cambridge Structural Database

**DOI:** 10.1107/S2053229626008417

**Published:** 2026-09-05

**Authors:** Florian Meurer

**Affiliations:** a Faculty for Chemistry and Pharmacy, University of Regensburg, Universitätsstrasse 31, Regensburg, 93053, Germany; University of North Texas at Dallas, USA

**Keywords:** Cambridge Structural Database, CSD, crystallographic validation, refinement indicators, residual electron density, small-mol­ecule crystallography

## Abstract

In this article, the temporal progression of quality indicators in the Cambridge Structural Database is evaluated statistically as a simplistic and accessible measure for structural quality trends. *R*1 showed a strong decrease until 1980 followed by com­paratively small changes, whereas *wR*2 increased significantly between 2000 and 2025. Residual electron-density extrema remained broadly stable, although the magnitude of the residual densities showed a small significant decrease. Exploratory normalization by unit-cell volume produced decreasing recent *R*1 and *wR*2 ratios, whereas *F*(000)-normalized *R*1 decreased and *F*(000)-normalized *wR*2 remained stable. Also, quality indicators are plotted on an elemental basis, and their trends are described.

## Abstract 2

A statistical evaluation of the entries in the Cambridge Structural Database reveals trends in structural quality as described by the accessible quality indicators in the CSD with respect to the structures’ publication year and the elements contained.

## Introduction

Single-crystal structure determination by X-ray, neutron or electron diffraction is the gold standard for qualitative analysis (Bragg & Bragg, 1913 ▸; Ewald, 1962 ▸; Authier, 2013 ▸). With a rich and successful history, structural scientists can look back at over 110 years of development, advances and applications. As far as data management is concerned, a cornerstone for small-mol­ecule structures was the foundation of the Cambridge Structural Database (CSD) by Olga Kennard in 1965 (Allen, 2002 ▸; Groom *et al.*, 2016 ▸; Hargittai & Hargittai, 2023 ▸). The CSD quickly became the central home of curated and machine-readable structural data. Today, with about 1.4 million entries, the CSD is one of the largest curated scientific databases in the world (Taylor & Wood, 2019 ▸). With a modern software toolkit for structural analysis (Bruno *et al.*, 2002 ▸; Allen & Bruno, 2010 ▸), the database is versatile and useful in various fields employing structural science, including small-mol­ecule chemistry, structural chemistry, crystal engineering, pharmaceutical chemistry, com­putational chemistry, materials science, quantum crystallography, machine-learning and data-driven modelling, and educational applications (Battle *et al.*, 2010 ▸; Allen & Bruno, 2010 ▸; Pallikara *et al.*, 2024 ▸; Stuke *et al.*, 2020 ▸). This is especially true in small-mol­ecule crystallography, where CSD-based tools provide duplicate checks for newly determined structures, while the CSD deposition workflow incorporates structural validation through the IUCr *checkCIF*/*PLATON* service (Bruno *et al.*, 2002 ▸; Spek, 2020 ▸). The database further supports systematic analyses of mol­ecular conformations, inter­molecular inter­actions, crystal packing, crystal engineering and polymorphism.

Single-crystal structure determination has undergone numerous significant improvements, both from a technological and a software perspective. For instance, X-ray radiation sources have experienced a massive improvement: modern rotating-anode sources and metal-jet technology now produce above 10^11^–10^13^ photons s^−1^ (Hemberg *et al.*, 2003 ▸; Graw *et al.*, 2023 ▸), bringing them to the same level as third-generation synchrotron facilities (Lindley, 1999 ▸). On the other hand, fourth-generation modern synchrotron devices can achieve fluxes of up to 10^13^–10^15^ photons s^−1^ (Eriksson *et al.*, 2014 ▸; Raimondi *et al.*, 2023 ▸; Schroer *et al.*, 2018 ▸), making radiation damage an increasingly significant problem in small-mol­ecule crystallography (Christensen *et al.*, 2019 ▸). Photon detection technologies for X-rays have seen stepwise improvement as well, from point detectors, which were very accurate, but needed a long time and a stable sample for each experiment, to charge-coupled devices (CCD) (Gruner & Ealick, 1995 ▸), which made data acquisition much faster, to ‘the best of both worlds’, modern photon counting devices, detectors that are fast and able to detect ‘single photons’ (Broennimann *et al.*, 2006 ▸; Johnson *et al.*, 2014 ▸).

Theory, software and modelling techniques were similarly improved by incorporating the Debye–Waller factor (Debye, 1913 ▸; Waller, 1923 ▸) into the crystallographic model, direct methods for structure solution (Karle & Hauptman, 1950 ▸), modern integration and processing techniques (Duisenberg *et al.*, 2003 ▸), absorption correction (Blessing, 1995 ▸; Krause *et al.*, 2015 ▸) and charge density/quantum crystallographic models (Coppens & Hermansson, 1998 ▸; Grabowsky *et al.*, 2017 ▸; Kleemiss *et al.*, 2021 ▸). Computer programs for data collection and processing, such as *XDS* (Kabsch, 2010 ▸), *EVAL* (Duisenberg *et al.*, 2003 ▸), *CrysAlis PRO* (Rigaku OD, 2025 ▸) and *APEX* (Bruker, 2025 ▸), and modern structure solution and refinement programs, such as the *SHELX* suite (Sheldrick, 2015*a* ▸; Sheldrick, 2015*b* ▸), *ShelXle* (Hübschle *et al.*, 2011 ▸), *OLEX2* (Bourhis *et al.*, 2015 ▸; Dolomanov *et al.*, 2009 ▸) and *JANA* (Petříček *et al.*, 2023 ▸), have also seen vast improvements in speed and sophistication.

Recently, Tovee *et al.* (2025 ▸) published a survey of crystallographic quality metrics in the CSD. The authors analysed *R* factors, max/min residual electron-density peaks, goodness-of-fit values, parameter shifts and resolution, com­pared the results across several structure classes and provided a perspective on future adaptations with respect to machine-learning and data-mining com­patibility.

Complementary to the above survey, this work examines the temporal evolution of accessible quality parameters for structures deposited in the CSD through the end of 2025. The chosen parameters were *R*1, which has the most extensive coverage across the whole CSD data set, and, additionally, *wR*2 and the minimum and maximum residual electron-density peaks, which have substantial coverage only in this millennium. *R*1 provides insight into the agreement between the measured and modelled/calculated structure factors, while *wR*2 describes the weighted agreement between squared measured and modelled/calculated structure factors. Minimum and maximum residual densities com­plement the two relative agreement factors with a third absolute value, describing the peaks in the difference Fourier map between the observed and calculated electron densities. Cell volume and *F*(000) are considered as relatively coarse measures of structural com­plexity, and its implications for structural quality are evaluated.

Throughout this scientific commentary, the term *structural quality* is used in an operational and deliberately restricted sense, referring to the reported agreement between the diffraction data and the refined structural model, as repre­sent­ed by *R*1, *wR*2 and the extrema of the residual electron density. These indicators do not com­prehensively describe structural correctness, precision or suitability for a particular application, but constitute widely reported and database-accessible descriptors suitable for a CSD-wide statistical com­parison.

## Data processing and description

For this work, a dataset was generated based on the CSD entries available *via* the CSD Python API (Version 3.6.2) as of 14 February 2026. The dataset contained 1394755 entries, which were deposited up to the end of 2025. Table 1 ▸ summarizes the CSD-extracted fields used for the analyses.

For the residual electron-density fields, values with absolute magnitude above 50 e Å^−3^ were excluded before summary statistics were com­puted. In addition, refinement_residual_electron_density_min (min) was restricted to values ≤ 0, refinement_residual_electron_density_max (max) was restricted to values ≥ 0 and cell_volume was restricted to values > 0. *R*1 was restricted to values ≤ 50% and refinement_weighted_r_factor (*wR*2) was restricted to values ≤ 75%. This removed 524 entries from cell_volume, 27 entries from *R*1, 199 entries from *wR*2, 576 entries from min and 532 entries from max.

The full CSD entries are heavily influenced by X-ray single-crystal data sets. According to Tovee *et al.* (2025 ▸), only 0.11% of the CSD contain structures measured with non-X-rays. Also, 31.2% of all structures show disorder, 13.0% are polymeric and only 0.17% use nonspherical atomic form factors. For the present analysis, these proportions mean that the results in this study are mostly based on IAM X-ray single-crystal structures, approximately one third of which contain disorder in the structural model. No entries were excluded on the basis of diffraction method, disorder, polymeric character, modulation, twinning, redetermination or refcode-family membership.

## Results and discussion

### Temporal evolution

As a first approach to answer the title question, it was informative to look at the progression of the quality indicators *R*1, *wR*2, and |min|/max residual electron-density peaks over time. Fig. 1 ▸ shows these developments alongside a linear fitting model. For *R*1, there is a clear break point around 1980. Therefore, separate linear fits describe the time before and after the year 1980. Since the *wR*2 is covered consistently only in the more recent years, the linear fit covers the range of data from 2000 to 2025. Because the average data points for the residual electron-density descriptors were highly variable, their fits were also restricted to the time span between 2000 and 2025. Because of a trend change, the partial fits between 2000–2013 and 2013–2025 are also discussed for the residual density peaks. A full description of the statistical fits can be found in Table S1 in the supporting information.

Publication year is used as the consistently accessible temporal descriptor. It may include post-date data collection and structure refinement, particularly for early entries, and therefore should not be inter­preted as the exact date at which the underlying experimental or refinement methodology was applied.

The progression of *R*1 showed a strong decrease with a slope of −0.41% per year until the year 1980, where an average value of 5.92% was reached. Afterwards, the *R*1 flattened out with a slope of −6.79 × 10^−3^% per year. An average *R*1 value of 5.37% was finally reached for 2025. The progression in *R*1 indicated only a small decrease after 1980, with an increasing error, and thus more diverging values, but still centred around the post-1980 average of 5.17% (slightly lower than the global average of 5.21%). In contrast, the *wR*2 showed a steady increase, indicating a worsening of the weighted agreement between measured and calculated squared structure factors. Here, the fit described a low 0.05% per year increase.

The maximum residual density peaks showed a trend reversal in 2013. Before 2013, max systematically increased, with a slope of 2.38 × 10^−3^ e Å^−3^ per year. After 2013, a decrease of −3.24 × 10^−3^ e Å^−3^ per year led to an overall decrease by −2.30 × 10^−4^ e Å^−3^ per year in the period between 2000 and 2025. A similar trend was observed for the absolute minimum residual densities. Here, the overall trend between 2000 and 2025 showed decreasing |min| densities, with a slope of −3.38 × 10^−4^ e Å^−3^ per year, with the segmented fits to and from 2013 being statistically insignificant at a significance level of 0.05. The temporal progression shows that the asymmetry of the absolute averages, which was also described by Tovee *et al.* (2025 ▸), has been a stable feature in the database since at least the beginning of the millennium, after which this statistic was consistently included in the entries.

Most importantly, these trends describe a generally high data–model agreement and a substantial historical improvement. For a timespan of over 40 years, the trends describe a combination of improvements in technology and software, and the increased tractability of com­plicated crystal structures. The most recent trends show a slow increase and a slow decrease in structural quality in *R*1 and *wR*2, respectively. The absolute trend is more pronounced in the weighted measure *wR*2 and in *R*1. The asymmetry of average min and max densities also shows that, on average, in the entire CSD, there is an excess of positive or unexplained residual electron density. Alongside systematic or artificial errors, this residual electron density should also capture the shortcomings of the independent atom model, which does not describe bonding density explicitly and may therefore contribute to part of the observed residual density, particularly for suitable high-quality data. This could potentially be improved by a more routine use of quantum crystallographic methods, such as Hirshfeld atom refinement or multipole/database approaches (Jayatilaka & Dittrich, 2008 ▸; Capelli *et al.*, 2014 ▸; Hansen & Coppens, 1978 ▸; Pichon-Pesme *et al.*, 1995 ▸). Nonspherical atomic form factors have become increasingly accessible in recent years through software developments such as *NoSpherA2* and *DiSCaMB* (Kleemiss *et al.*, 2021 ▸; Chodkiewicz *et al.*, 2018 ▸). However, multiple effects across the database such as absorption, disorder, diffuse or overlooked solvent content, twinning, significant multiple scattering and problematic heavy atoms – especially with respect to anomalous dispersion corrections – probably all contribute to the asymmetric and total amount of residual electron density in the structures in the CSD.

A possible descriptor of increasing com­plexity in crystal structures is the cell volume, since in general, larger mol­ecules or larger unit cells lead to greater com­plexity in both the data and the model. As an approximation, this approach assumes that other possible measures for structural com­plexity, such as the content of non-H-atoms, *Z*′, the number of refined parameters, appearance or modelling of disorder, (diffuse) solvent content or heavy-atom content, are uniformly distributed. This assumption cannot be checked with the current accessible data *via* the CSD Python API. For this study, cell-volume- and *F*(000)-normalized quality indicators were calculated and fitted with a linear fit (Fig. 2 ▸). *F*(000) values are not obtained from the CSD extraction, but calculated from the sum formula by the equation
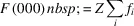
where *Z* is the number of formula units in the unit cell and *f_i_* is the elastic atomic form factor value at the zero scattering angle, for each atom in sum formula *i*.

For the cell-volume-normalized *R*1 metric, the progression pre-1980 also showed a significant decrease with a slope of −1.16 × 10^−4^% Å^−3^ per year, while the *F*(000)-normalized *R*1 exhibited a decrease of −1.68 × 10^−4^% e^−1^ per year over the same period. Post-1980, both progressions continued to show decreasing trends, with the CV-normalized *R*1 declining at −1.61 × 10^−5^% Å^−3^ per year and the *F*(000)-normalized *R*1 at −3.93 × 10^−5^% e^−1^ per year. These trends indicate that the average cell volume and scattering power per structure, as proxies for structural com­plexity, increased faster than the ratio of measured and modelled structure factors.

For the *wR*2 metric, normalization approaches revealed contrasting trends across different time periods. Over the full time span (1963–2025), the CV-normalized *wR*2 showed minimal change with a slope of 1.39 × 10^−5^% Å^3^ per year, while the *F*(000)-normalized *wR*2 displayed a slight increase of 7.23 × 10^−5^% e^−1^ per year. However, the post-2007 period revealed a distinct reversal: the CV-normalized *wR*2 decreased at a rate of −7.52 × 10^−6^% Å^−3^ per year, whereas the *F*(000)-normalized *wR*2 showed negligible and insignificant change with a slope of −2.78 × 10^−6^% e^−1^ per year. This transition suggests that recent refinement improvements have been decoupling from increases in cell volume and atomic content, with *wR*2 improvements now becoming more pro­noun­ced relative to structural com­plexity as measured by cell volume.

In summary, the CSD temporal data describe an incongruity between *R*1 and *wR*2. While *R*1 decreased until *ca* 1980 and then remained stable to the present day, *wR*2 rose slowly but significantly every year, indicating a lower agreement between the weighted measured and calculated structure factors on average. When the cell volume and the scattering power of the unit cell were taken into account as an indicator of structure com­plexity, the trend in normalized *wR*2 was reversed, as *wR*2 decreased concomitantly with the normalized *R*1.

### Elemental data

Another question that was addressed by the examination of this CSD data set was where in the Periodic Table (PT) problematic regions were located, and which elements were associated with higher or lower average indicator values. These results are to be taken with caution, as they only represent the statistical mean per element. The data shown do not account for elemental co-occurrence or the low number of entries available for some elements.

Fig. 3 ▸ shows the average *R*1 and *wR*2 values for each element in the PT.

In general, there is a good agreement between the elemental values for *R*1 and *wR*2. Carbon-containing structures have *R*1 and *wR*2 values of 5.22 and 13.47%, respectively, which are very close to the global averages for *R*1 and *wR*2, consistent with most structures in the CSD containing carbon. Notably, the heavier halogens Br and I, which are notorious for absorption issues and artificial residual electron densities, nevertheless show lower-than-average, and thus better, quality indicators.

These trends indicate that, with increasing atomic number, the quality indicators *R*1 and *wR*2 improve, while |min| and max densities increase. For *R*1 and *wR*2, the trend shows that with increasing scattering power, structures are more clearly defined by the heavy atoms, their positions and ADPs. These two relative quality indicators are less affected by small inaccuracies, *e.g.* due to disorder or other crystallographic challenges, than by the heavy-atom atomic parameters. Also implicated in this trend is that for lighter elements, bonding density, which is uncaptured by the default independent atom model, makes up a higher percentage of the total electron density or scattering power. This effect further worsens the relative quality indicators for lighter atomic structures. To improve quality indicators for lighter-element structures, a non-spherical atomic form factor could provide significant benefits across the CSD and PT.

The |min| and max residual electron density, however, indicate in absolute terms the features not modelled in the crystal structure. As *Z* rises, these absolute numbers increase as well and a better treatment of artificial effects, such as absorption correction or anomalous dispersion correction, becomes more and more necessary for heavier element structures.

Fig. 4 ▸ shows the PT colour-coded by |min| and max residual electron density. A surprising outlier is boron, which could indicate high com­plexity in chemical bonding, which is again not fully captured by the independent atom model, or general crystallographic problems such as poorer data–model agreement or greater modelling com­plexity in boron-containing entries. A similar pattern is observed to a lesser degree with fluorine, sulfur and the alkali metals, except for lithium. Tungsten is notable for com­paratively large mean residual-density extrema.

## Conclusion

In conclusion, this work presents the temporal evolution of selected quality indicators in the CSD, which com­prises almost 1.4 million structures as of the end of 2025. The trends show a significant increase in *wR*2 and com­paratively small changes in *R*1 after its strong historical decrease, indicating an influence of either the difference between intensities and structure factors or the employed weighting scheme. When cell volume is taken into account as an index of structural com­plexity, especially the cell volume normalized *wR*2 and normalized *R*1 show decreasing ratios over at least the last 19 years. *F*(000)-normalized *R*1 decreased, whereas no significant post-2007 trend was detected for *F*(000)-normalized *wR*2. A com­parison of |min| and max residual electron densities reveals the stable, asymmetric temporal progression of these residuals, possibly attributable to the shortcomings of the independent atom model.

When quality indicators were analysed on an elemental basis, the trends observed were an increase in |min| and max residual electron density and an improvement in relative quality indicators *wR*2 and *R*1 as functions of atomic number. These trends in both the relative and absolute measures were as expected for crystallographic reasons. Part of the trend toward poorer *R*1 and *wR*2 values for low-*Z* structures could also be explained by shortcomings of the independent-atom model (IAM), while high |min| and max densities for heavy elements could be improved by better treatment of their relativistic, absorption and anomalous dispersion effects.

This article provides a CSD-wide perspective on how com­monly reported crystallographic quality indicators are used, what they represent, and which experimental and modelling factors influence them. Their temporal statistical evaluation provides insight into the development of structural quality in the reported structures.

## Supplementary Material

Data collection and processing; Temporal-quality analysis; Elemental-average analysis; AI statement; Plotted data. DOI: 10.1107/S2053229626008417/yd3074sup1.pdf

## Figures and Tables

**Figure 1:**
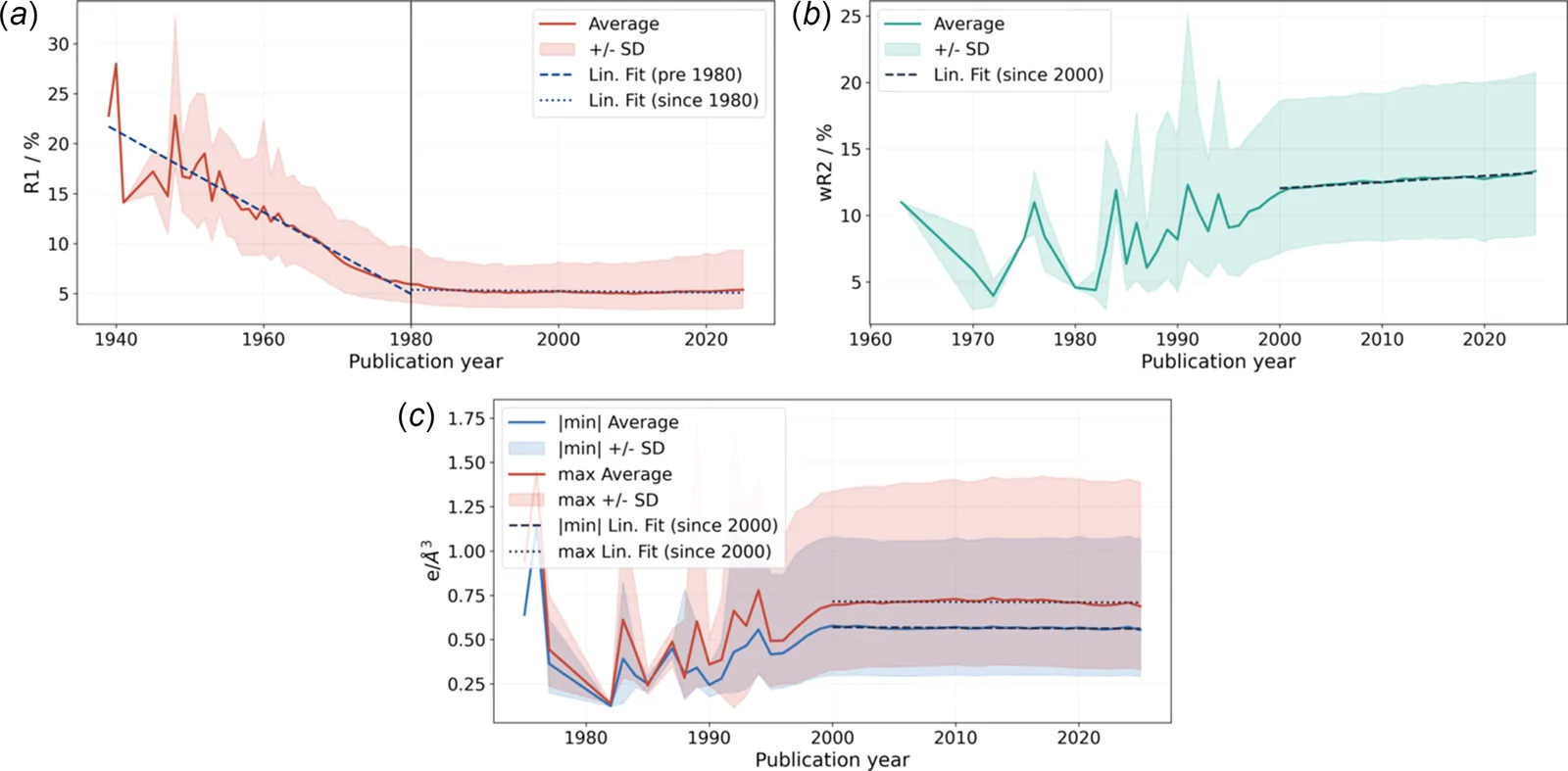
Progression of average (*a*) *R*1, (*b*) *wR*2 and (*c*) |min|/max residual electron-density peaks over time, with asymmetric standard deviations around the average value and a linear fit. Fit statistics can be found in Table S1 in the supporting information.

**Figure 2:**
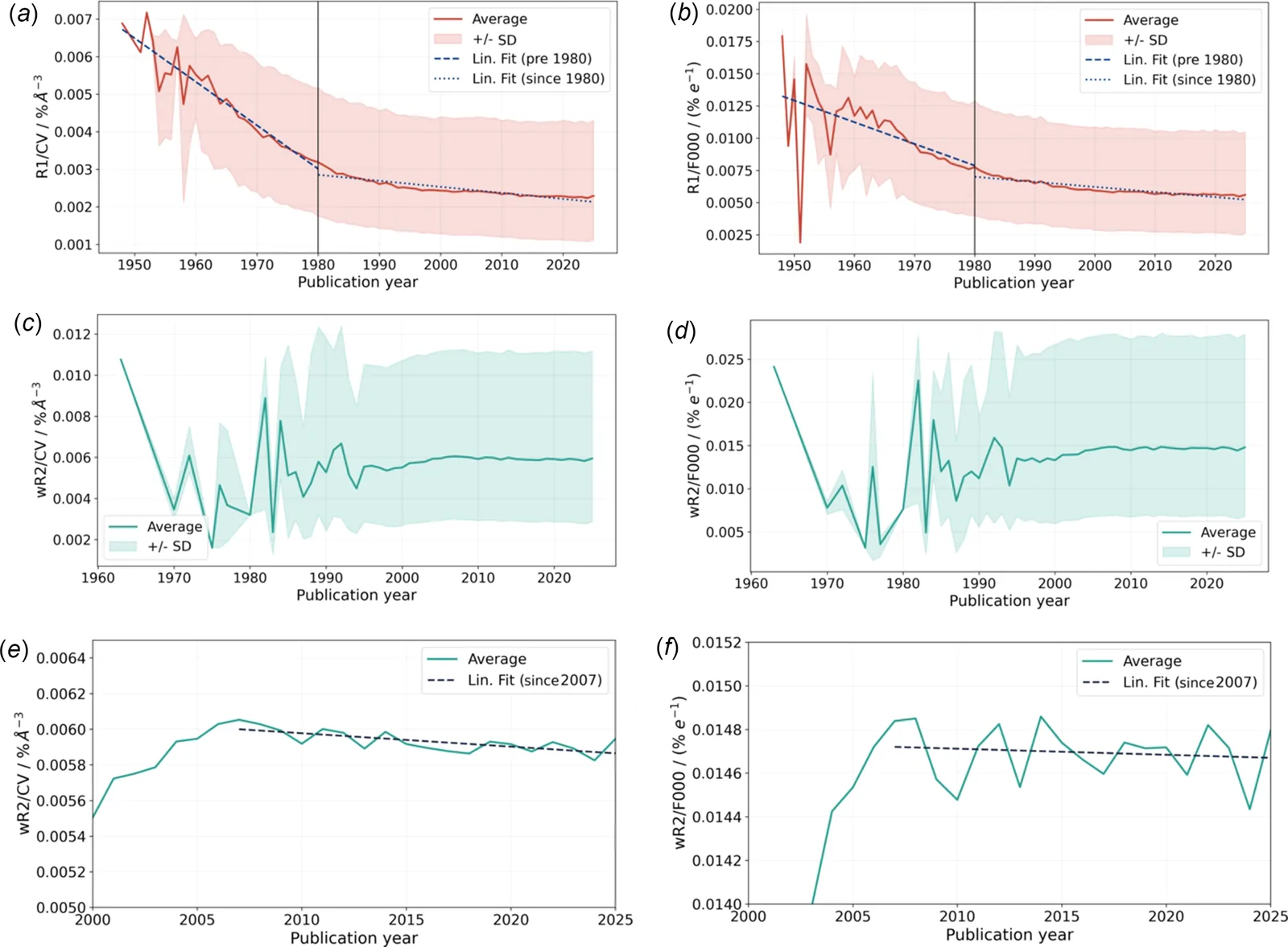
Progression over time of the cell-volume-normalized (left) and *F*(000)-normalized (right) average (*a*)/(*b*) *R*1 and (*c*)/(*d*) *wR*2, with asymmetric standard deviations around the average value. (*e*)/(*f*) A zoomed-in region of panels (*c*)/(*d*), with a linear fit from publication year 2007 onwards.

**Figure 3:**
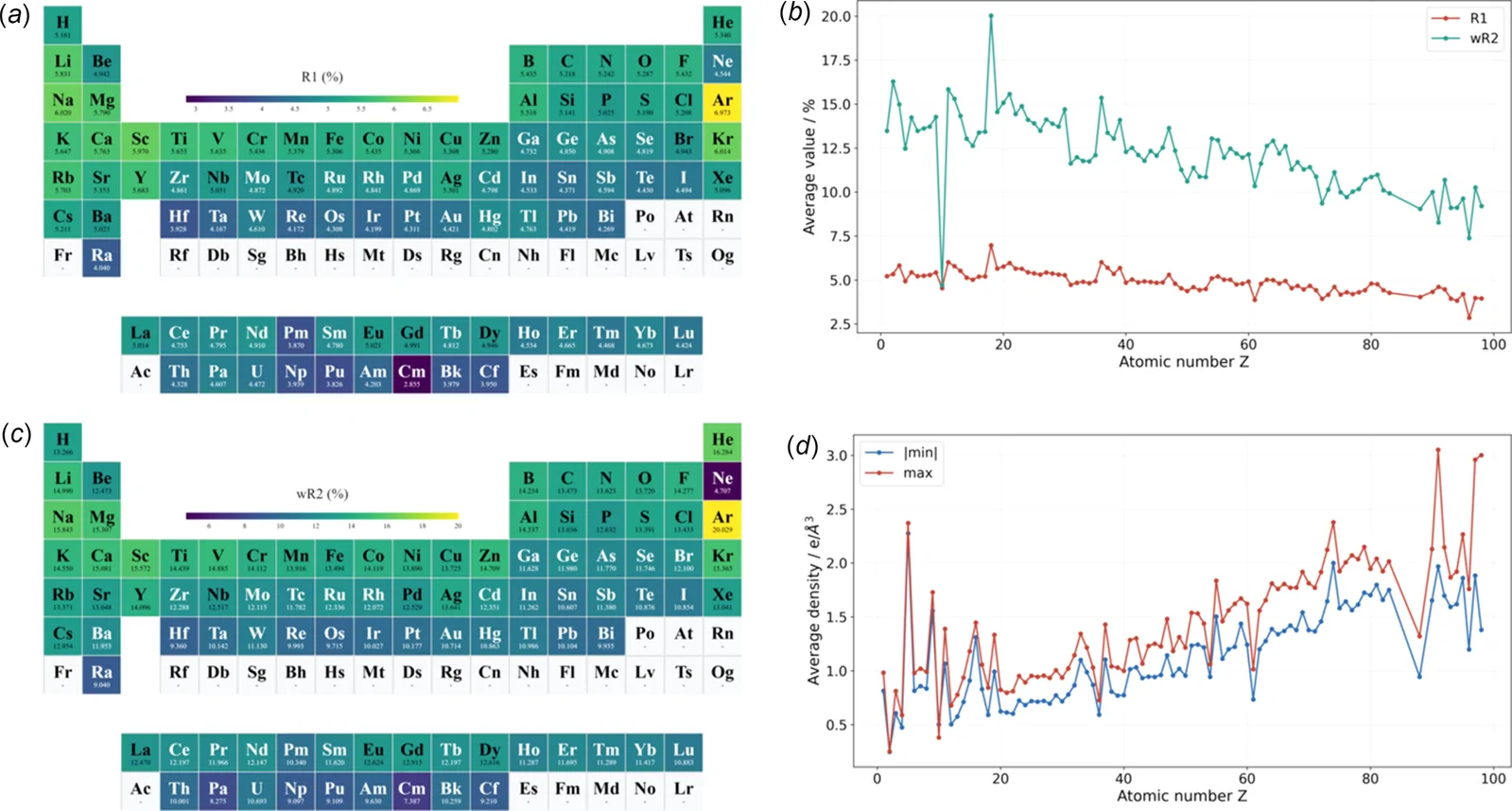
Average (*a*) *R*1 and (*c*) *wR*2 values colour coded by element. (*b*) Average *R*1 and *wR*2 and (*d*) |min| and max residual electron densities as a function of atomic number *Z*. Averages are calculated on an occurrence basis, rather than weighted by their stoichiometric coefficient in a given sum formula. Neon (*Z* = 10) deviates from the general trend because it has only two usable entries in the CSD.

**Figure 4:**
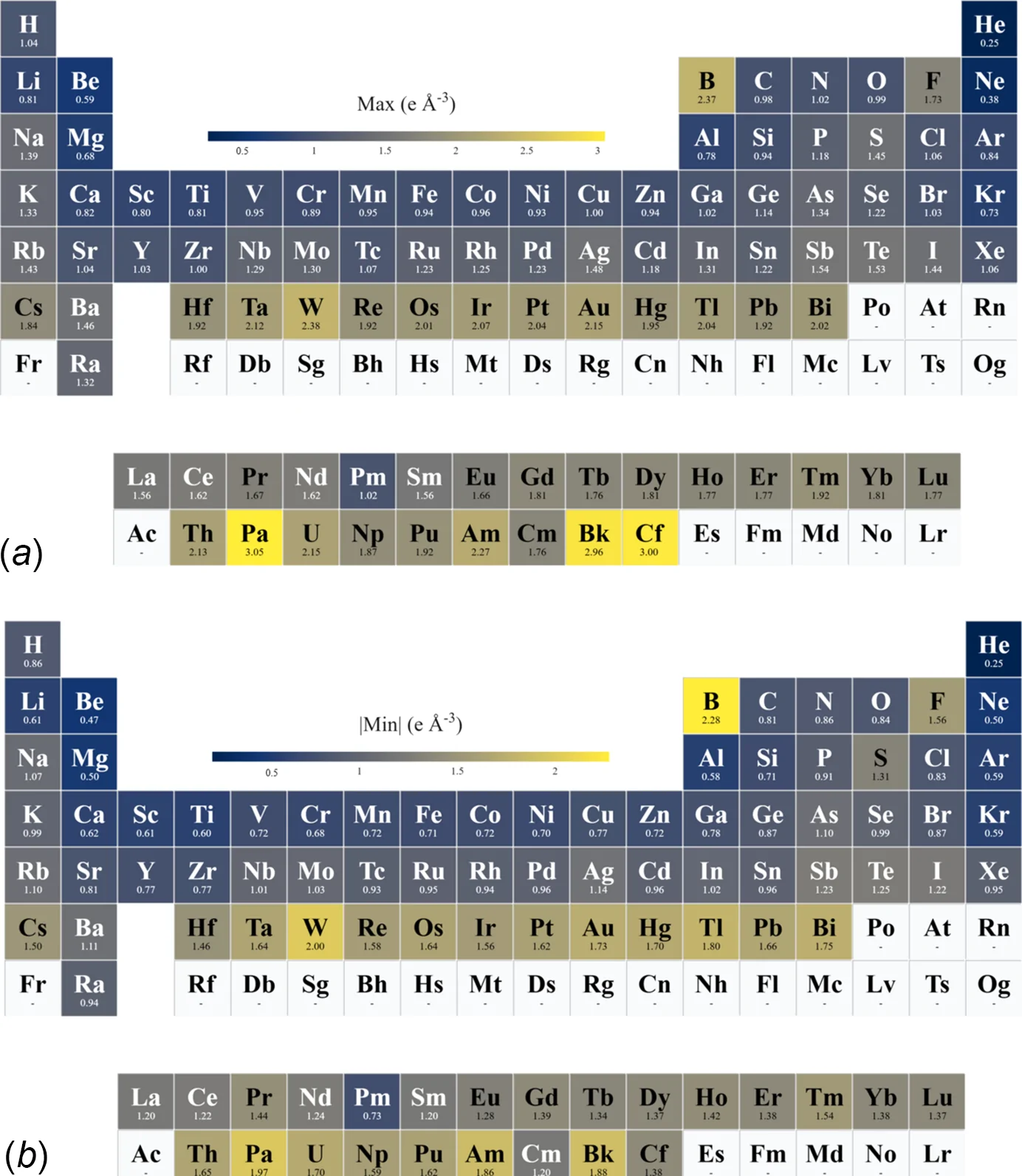
Average (*a*) max and (*b*) |min| residual electron-density values per element in the Periodic Table. Averages are calculated on an occurrence basis, rather than weighted by their molar frequency in a given sum formula.

**Table 1:** Data fields extracted from the CSD for this work, the number of entries and statistical descriptors for each field *N* gives the number of entries in the dataset, ‘Name’ refers to the name used within the article and SD gives the standard deviation.

Field	Name	*N*	Mean	Min	Max	SD
formula	–	1393581	NA	NA	NA	NA
publication_year	–	1394719	2010.2	1923	2025	11.883
cell_volume (Å^3^)	–	1394231	3490.1	15.244	11950873	19831
r_factor (%)	*R*1	1373754	5.2133	0.026	49.24	2.5071
refinement_weighted_r_factor (%)	*wR*2	1068869	13.416	0.19	74.98	7.1756
refinement_residual_electron_density_min (e Å^−3^)	min	1063997	−0.7057	−49.432	−0.001	0.74045
refinement_residual_electron_density_max (e Å^−3^)	max	1064555	0.89118	0.001	49.51	0.984

## Data Availability

The data used herein were retrieved from the Cambridge Structural Database (Version 6.01) *via* the CSD Python API (Version 3.6.2) and the site CSD license (ID 2068) of the University of Regensburg.
