# Supplementary material for: Are crystal structures getting better? A temporal and elemental analysis of the Cambridge Structural Database
Source: Acta Crystallogr C Struct Chem. 2026 Sep 5;82(Pt 10):603–9. doi: 10.1107/S2053229626008417 (PMC13637800; doi:10.1107/S2053229626008417)
Supplement: Supplementary file 1 [file c-82-00603-sup1.pdf]

STRUCTURAL CHEMISTRY

Volume 82 (2026)

Supporting information for article:

Are crystal structures getting better? A temporal and elemental analysis of the Cambridge Structural Database

Florian Meurer

S1. Data collection and processing

S1.1. CSD Python API CSV generation.

The CSV datasets used in this study were generated directly from the CSD Python API. Extraction scripts iterated over EntryReader('CSD').entries() and wrote one row per structure to a CSV file. The main export included identifiers, formula, publication metadata, crystal properties, refinement metrics, and experimental information. In particular, the extracted fields included refcode, accession number, formula, cell parameters and volume, calculated density, space group descriptors, R factors, weighted R factors, goodness of fit, residual electron-density extrema, reflection counts, radiation metadata, and publication year.

- The comprehensive CSV export was implemented in scripts such as enhanced_csd_extraction.py and fetch_csd_comprehensive.py.
- Chunked exports csd.part*.csv were used for downstream analyses to keep memory use manageable.
- The filtering utility analyse_csd.py wrote csd_filtered.csv from the CSD API after applying structure-level criteria.
- The temporal analysis scripts read the part files and derived time-dependent statistics from the exported columns.

S1.2. Derived datasets

The downstream analysis produced yearly trend tables, per-element average datasets, and periodic-table/Z-axis plots. Element-level metrics were computed from parsed molecular formulas and aggregated by element presence. For the *Z*-axis plots, each element symbol was mapped to its atomic number and plotted in natural periodic order.

S2. Temporal-quality analysis

S2.1. Quality indicator coverage in the CSD by year

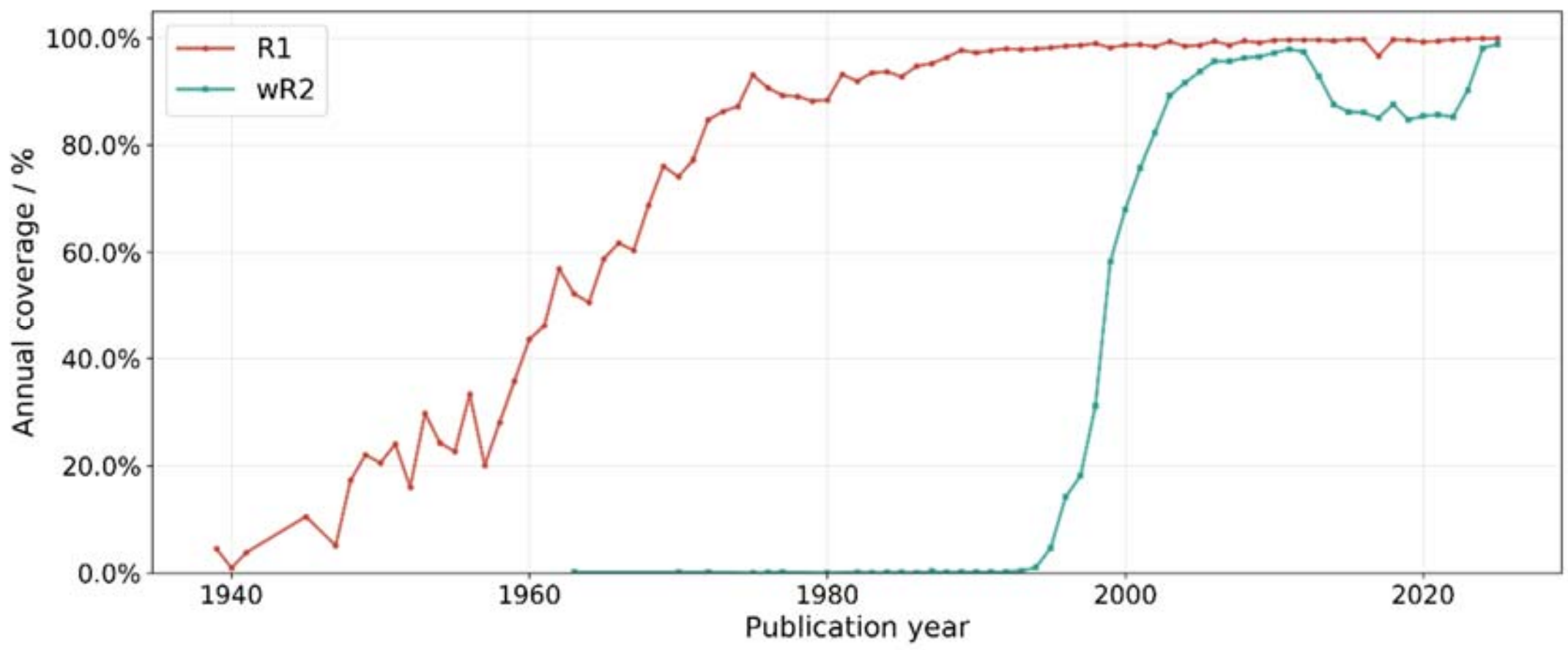

Figure S1 Annual coverage in % of the quality indicators R1 and wR2 in the extracted CSD data.

The increase in wR2 coverage around the turn of the millennium coincides with the widespread adoption of refinement against F^2using all measured data. Nevertheless, reported wR2 values remain dependent on the applied weighting scheme and refinement program and may therefore not be strictly uniform across all CSD entries.

S2.2. Calculation of asymmetric standard deviation

The asymmetric standard deviation (referred to as SD) was calculated as

$$\text{sd\_plus} = \sqrt{\frac{1}{n_+}\sum_{x \geq \bar{x}} (x - \bar{x})^2}$$

$$\text{sd\_minus} = \sqrt{\frac{1}{n_-}\sum_{x < \bar{x}} (\bar{x} - x)^2}$$

S2.3. Fitting parameters

Table S1 Overview of the linear fit statistics in Figures 1 and 2.

Metric	Lin. fit	Year min.	Year max.	N	Slope	R^2	p-value
R1	≤1980	1939	1980	38	-0.409	0.845	3.60×10^{-16}
R1	≥ 1980	1980	2025	46	-0.00679	0.205	0.00156
wR2	≥ 2000	2000	2025	26	0.0465	0.907	7.29×10^{-14}
R1/CV	≤ 1980	1948	1980	31	-1.16×10^{-4}	0.904	2.68×10^{-16}
R1/CV	≥ 1980	1980	2025	46	-1.61×10^{-5}	0.821	4.58×10^{-18}
wR2/CV	≥ 2007	2007	2025	19	-7.52×10^{-6}	0.493	8.07×10^{-4}
R1/F(000)	≤ 1980	1948	1980	33	-1.68×10^{-4}	0.306	8.41×10^{-4}
R1/F(000)	≥ 1980	1980	2025	46	-3.93×10^{-5}	0.833	9.79×10^{-19}
wR2/F(000)	≥ 2007	2007	2025	19	-2.78×10^{-6}	0.148	0.620
max density	2000	2000	2025	26	-2.30×10^{-4}	0.0220	0.469
max density	≤ 2013	2000	2013	14	2.38×10^{-3}	0.798	1.67×10^{-5}
max density	≥ 2013	2013	2025	13	-3.24×10^{-3}	0.805	3.22×10^{-5}
|min| density	2000	2000	2025	26	-3.38×10^{-4}	0.210	0.0186

Metric	Lin. fit	Year min.	Year max.	N	Slope	R^2	p-value
|min| density	≤ 2013	2000	2013	14	-5.71×10^{-4}	0.166	0.148
|min| density	≥ 2013	2013	2025	13	-7.45×10^{-4}	0.288	0.0585

S3. Elemental-average analysis

Average metrics versus atomic number. Average R1 and wR2 were plotted against atomic number *Z*, together with average |min| and max residual densities. The R1/wR2 panel includes horizontal reference lines for the global averages, and the wR2 periodic-table plot was also generated without Ne as a sensitivity check.

Table S2 Correlations of elemental averages with the atomic number *Z*.

metric	n	pearson_r	spearman_rho
R1	93	-0.766414	-0.808361
wR2	93	-0.708099	-0.810973
abs_min_density	93	0.733300	0.771180
max_density	93	0.795960	0.817388

S4. AI statement

For the data analysis and dataset generation, the GitHub Copilot in the Microsoft Visual Studio Code Editor was used.

Role of the AI assistant. An AI coding assistant (GPT-5.4 mini) was used for the CSV-generation and analysis scripts, collate the plotting outputs, and draft this Supporting Information document in a concise IUCr-style format. The assistant also generated contents of the supporting information from these outputs.

The author has reviewed all output and code that the AI provided for consistency.

S5. Plotted data

S5.1. Fig 1 A

year	mean	sd_minus	sd_plus	*N*
1939	22.800	0.000	0.000	1
1940	28.000	0.000	0.000	1
1941	14.100	0.000	0.000	1
1945	17.200	2.267	2.025	4
1947	14.750	3.889	3.889	2
1948	22.822	5.207	10.000	9
1949	16.713	3.613	3.671	15
1950	16.547	4.007	7.435	15
1951	18.016	6.522	7.091	19

1952	19.020	6.495	5.924	21
1953	14.293	4.142	5.277	42
1954	17.228	4.156	4.427	33
1955	15.072	4.275	5.881	29
1956	14.511	3.982	5.411	48
1957	13.403	4.589	5.082	38
1958	13.465	4.614	4.980	57
1959	12.442	3.650	6.381	67
1960	13.723	4.729	8.726	102
1961	12.197	3.861	4.696	133
1962	13.005	4.027	6.639	156
1963	11.770	4.097	4.783	219
1964	11.777	3.750	5.112	243
1965	11.184	3.437	4.722	386
1966	10.806	3.489	4.908	426
1967	10.492	3.610	5.095	569
1968	9.926	3.482	4.335	672
1969	9.256	3.330	4.342	863
1970	8.592	3.052	3.717	941
1971	8.031	2.938	4.385	1200
1972	7.636	2.684	4.646	1678
1973	7.354	2.630	4.308	1721
1974	7.081	2.454	4.480	1871
1975	6.774	2.302	3.971	2029
1976	6.485	2.082	3.819	2494
1977	6.238	1.954	3.444	2768
1978	6.258	1.992	3.459	3050
1979	6.035	1.915	3.778	3428
1980	5.925	1.835	3.525	3764
1981	5.903	1.865	3.609	4350
1982	5.659	1.743	3.130	4820
1983	5.555	1.708	2.989	4911
1984	5.457	1.680	3.098	6106
1985	5.374	1.613	2.826	6424
1986	5.338	1.601	2.858	6516
1987	5.298	1.636	2.888	7144
1988	5.198	1.591	2.864	7420
1989	5.210	1.558	2.733	7648
1990	5.130	1.515	2.670	8721
1991	5.182	1.563	2.847	9744
1992	5.169	1.541	2.841	10527
1993	5.088	1.521	2.646	11825
1994	5.136	1.524	2.663	12059
1995	5.099	1.525	2.702	12797
1996	5.139	1.526	2.656	15387
1997	5.151	1.541	2.774	15760
1998	5.183	1.559	2.735	17195

1999	5.177	1.568	2.757	18593
2000	5.235	1.590	2.934	19839
2001	5.185	1.575	2.836	21648
2002	5.136	1.575	2.971	24104
2003	5.109	1.565	2.962	26287
2004	5.090	1.545	2.838	27853
2005	5.098	1.587	3.048	31523
2006	5.069	1.587	2.982	34739
2007	5.027	1.574	2.942	36419
2008	5.058	1.609	2.937	37107
2009	5.025	1.595	3.075	41193
2010	4.992	1.596	3.031	42306
2011	5.023	1.599	3.124	44865
2012	5.085	1.645	3.174	48022
2013	5.059	1.658	3.188	48888
2014	5.111	1.689	3.287	51827
2015	5.127	1.686	3.226	55074
2016	5.209	1.750	3.526	56741
2017	5.181	1.747	3.480	57573
2018	5.227	1.752	3.521	58096
2019	5.199	1.750	3.560	60847
2020	5.197	1.808	3.829	63447
2021	5.220	1.772	3.664	60269
2022	5.259	1.803	3.792	60456
2023	5.322	1.848	4.001	60434
2024	5.339	1.851	3.996	60176
2025	5.373	1.863	3.972	26951

S5.2. Fig 1 B

year	mean	sd_minus	sd_plus	*N*
1963	11.000	0.000	0.000	1
1970	5.920	2.998	2.998	2
1972	3.967	0.777	1.222	3
1975	8.230	0.000	0.000	1
1976	10.980	2.376	2.376	2
1977	8.426	2.617	2.190	5
1980	4.580	0.000	0.000	1
1982	4.393	0.600	1.534	7
1983	7.617	4.682	8.109	3
1984	11.900	1.981	1.981	4
1985	6.374	2.042	4.586	7
1986	9.438	4.290	8.427	4
1987	6.067	1.819	2.987	23
1988	7.293	2.819	8.857	15
1989	8.938	3.614	8.971	19
1990	8.192	3.913	7.786	20
1991	12.305	5.568	12.723	23

1992	10.361	4.469	7.495	27
1993	8.816	3.497	5.410	50
1994	11.599	5.042	8.780	121
1995	9.095	3.565	5.836	608
1996	9.235	3.809	5.868	2199
1997	10.299	4.055	5.851	2870
1998	10.587	3.985	6.311	5356
1999	11.239	4.406	6.623	10905
2000	11.733	4.567	6.895	13460
2001	12.049	4.585	6.736	16296
2002	12.075	4.467	6.666	19720
2003	12.142	4.379	6.707	23138
2004	12.259	4.378	6.576	25414
2005	12.341	4.406	6.611	29432
2006	12.359	4.389	6.576	32726
2007	12.479	4.384	6.573	34664
2008	12.577	4.447	6.657	35208
2009	12.526	4.391	6.640	39312
2010	12.471	4.403	6.684	40510
2011	12.560	4.414	6.734	43078
2012	12.754	4.502	6.858	45783
2013	12.738	4.533	6.898	44400
2014	12.843	4.567	7.029	44352
2015	12.786	4.497	6.950	46251
2016	12.834	4.579	7.053	47247
2017	12.825	4.600	7.045	49014
2018	12.921	4.573	7.143	49197
2019	12.848	4.549	7.083	49949
2020	12.737	4.698	7.260	52620
2021	12.891	4.577	7.246	50042
2022	12.977	4.631	7.231	49740
2023	13.014	4.655	7.347	52220
2024	13.172	4.719	7.392	56269
2025	13.329	4.775	7.420	25448

S5.3. Figure 1 C

year	abs_min_mean	max_mean
1975	0.640	0.940
1976	1.150	1.450
1977	0.363	0.443
1982	0.124	0.138
1983	0.391	0.612
1984	0.297	0.434
1985	0.250	0.240
1987	0.451	0.488
1988	0.306	0.284
1989	0.341	0.603

1990	0.244	0.359
1991	0.281	0.386
1992	0.429	0.661
1993	0.465	0.577
1994	0.555	0.777
1995	0.417	0.492
1996	0.423	0.494
1997	0.470	0.563
1998	0.524	0.623
1999	0.561	0.675
2000	0.577	0.696
2001	0.571	0.697
2002	0.576	0.708
2003	0.570	0.711
2004	0.563	0.704
2005	0.561	0.712
2006	0.560	0.713
2007	0.562	0.716
2008	0.563	0.718
2009	0.565	0.724
2010	0.570	0.729
2011	0.562	0.718
2012	0.563	0.718
2013	0.572	0.733
2014	0.565	0.721
2015	0.568	0.726
2016	0.562	0.719
2017	0.568	0.724
2018	0.567	0.718
2019	0.561	0.709
2020	0.568	0.710
2021	0.561	0.697
2022	0.557	0.692
2023	0.561	0.697
2024	0.571	0.709
2025	0.554	0.688

S5.4. Fig 2 A

year	mean	sd_minus	sd_plus	*N*
1948	0.0069	0.0000	0.0000	1
1951	0.0061	0.0000	0.0000	1
1952	0.0072	0.0000	0.0000	1
1953	0.0064	0.0003	0.0002	3
1954	0.0051	0.0015	0.0015	2
1955	0.0056	0.0017	0.0012	5
1956	0.0055	0.0017	0.0010	4
1957	0.0063	0.0001	0.0002	4

1958	0.0047	0.0026	0.0024	4
1959	0.0058	0.0021	0.0010	13
1960	0.0055	0.0011	0.0010	19
1961	0.0054	0.0019	0.0013	24
1962	0.0055	0.0021	0.0012	40
1963	0.0051	0.0021	0.0015	62
1964	0.0047	0.0017	0.0016	74
1965	0.0049	0.0018	0.0015	132
1966	0.0047	0.0018	0.0016	159
1967	0.0044	0.0019	0.0017	243
1968	0.0043	0.0017	0.0018	347
1969	0.0042	0.0018	0.0019	494
1970	0.0040	0.0016	0.0018	605
1971	0.0038	0.0016	0.0019	842
1972	0.0039	0.0016	0.0018	1214
1973	0.0038	0.0016	0.0019	1257
1974	0.0036	0.0015	0.0019	1454
1975	0.0036	0.0015	0.0020	1579
1976	0.0035	0.0015	0.0019	2087
1977	0.0034	0.0015	0.0019	2363
1978	0.0033	0.0014	0.0020	2595
1979	0.0032	0.0014	0.0020	3004
1980	0.0032	0.0014	0.0020	3297
1981	0.0031	0.0014	0.0019	3887
1982	0.0030	0.0014	0.0020	4369
1983	0.0029	0.0013	0.0020	4492
1984	0.0029	0.0013	0.0020	5675
1985	0.0028	0.0013	0.0020	6014
1986	0.0028	0.0013	0.0020	6083
1987	0.0028	0.0013	0.0020	6713
1988	0.0027	0.0012	0.0020	7015
1989	0.0027	0.0012	0.0020	7254
1990	0.0026	0.0012	0.0020	8286
1991	0.0026	0.0012	0.0020	9282
1992	0.0026	0.0012	0.0019	10057
1993	0.0025	0.0012	0.0019	11327
1994	0.0025	0.0012	0.0019	11543
1995	0.0025	0.0012	0.0019	12291
1996	0.0025	0.0011	0.0019	14761
1997	0.0024	0.0011	0.0020	15198
1998	0.0024	0.0011	0.0019	16547
1999	0.0024	0.0011	0.0019	17812
2000	0.0024	0.0012	0.0020	19021
2001	0.0024	0.0012	0.0020	20804
2002	0.0024	0.0012	0.0020	23081
2003	0.0024	0.0012	0.0020	25265
2004	0.0024	0.0012	0.0019	26775

2005	0.0024	0.0012	0.0020	30250
2006	0.0024	0.0012	0.0020	33315
2007	0.0024	0.0012	0.0020	35067
2008	0.0024	0.0012	0.0020	35682
2009	0.0024	0.0012	0.0020	39631
2010	0.0023	0.0012	0.0020	40771
2011	0.0024	0.0012	0.0020	43296
2012	0.0023	0.0012	0.0020	46363
2013	0.0023	0.0011	0.0020	47223
2014	0.0023	0.0012	0.0020	49963
2015	0.0023	0.0012	0.0020	53101
2016	0.0023	0.0012	0.0020	54598
2017	0.0023	0.0012	0.0020	55464
2018	0.0023	0.0012	0.0020	55854
2019	0.0023	0.0012	0.0020	58608
2020	0.0023	0.0012	0.0020	60686
2021	0.0023	0.0012	0.0020	57933
2022	0.0023	0.0012	0.0020	58216
2023	0.0023	0.0012	0.0020	57895
2024	0.0022	0.0012	0.0020	57706
2025	0.0023	0.0012	0.0020	25808

S5.5. Fig 2 B

year	mean	sd_minus	sd_plus	*N*
1948	0.017899	0.000647	0.000647	2
1949	0.009389	0.000000	0.000000	1
1950	0.014563	0.001835	0.001835	2
1951	0.001901	0.000000	0.000000	1
1952	0.015753	0.003901	0.003901	2
1953	0.014201	0.003054	0.002211	5
1954	0.012845	0.004926	0.002853	3
1955	0.011979	0.003296	0.002204	5
1956	0.008719	0.004235	0.005438	5
1957	0.012074	0.003252	0.001764	5
1958	0.012309	0.006334	0.004836	9
1959	0.013140	0.006059	0.003061	14
1960	0.011725	0.004175	0.003045	27
1961	0.012403	0.004368	0.004048	39
1962	0.011521	0.006039	0.004472	46
1963	0.012148	0.005009	0.003910	64
1964	0.010803	0.004567	0.004499	82
1965	0.011399	0.004746	0.004513	146
1966	0.011294	0.004331	0.004194	164
1967	0.010641	0.005370	0.005026	232

1968	0.010276	0.004590	0.004616	312
1969	0.009685	0.004434	0.004746	437
1970	0.009548	0.004500	0.004810	565
1971	0.008904	0.004086	0.004944	697
1972	0.008859	0.004157	0.004859	1025
1973	0.008740	0.004156	0.004884	1056
1974	0.008393	0.003997	0.005140	1233
1975	0.008413	0.003973	0.005203	1268
1976	0.008059	0.003796	0.004951	1673
1977	0.007902	0.003790	0.004938	1922
1978	0.007780	0.003619	0.004983	2001
1979	0.007593	0.003595	0.005012	2266
1980	0.007752	0.003779	0.005147	2464
1981	0.007460	0.003596	0.005054	2799
1982	0.007262	0.003531	0.005081	3168
1983	0.007090	0.003455	0.005040	3021
1984	0.007086	0.003399	0.004966	3776
1985	0.006877	0.003337	0.004948	4103
1986	0.006889	0.003356	0.005022	4006
1987	0.006669	0.003327	0.004993	4385
1988	0.006696	0.003295	0.005012	4448
1989	0.006703	0.003286	0.005016	4611
1990	0.006493	0.003156	0.004967	5198
1991	0.006648	0.003271	0.005054	5800
1992	0.006462	0.003195	0.004790	6378
1993	0.006240	0.003127	0.004926	7060
1994	0.006241	0.003114	0.004894	7347
1995	0.006222	0.003097	0.004866	7695
1996	0.006080	0.003066	0.004909	9192
1997	0.006016	0.003009	0.004910	9268
1998	0.006002	0.003041	0.004913	10075
1999	0.006026	0.003052	0.004801	10874
2000	0.005922	0.003062	0.004962	11820
2001	0.005899	0.003055	0.004896	13104
2002	0.005850	0.003055	0.005036	14637
2003	0.005787	0.003026	0.004963	16014
2004	0.005889	0.003051	0.004937	17073
2005	0.005846	0.003014	0.004922	19386
2006	0.005847	0.003018	0.004927	21555
2007	0.005841	0.003049	0.004897	22684
2008	0.005833	0.003018	0.004911	22729
2009	0.005729	0.002995	0.004967	25576

2010	0.005665	0.002966	0.004855	25861
2011	0.005716	0.003000	0.004896	27956
2012	0.005726	0.002991	0.004802	29880
2013	0.005581	0.002927	0.004874	29610
2014	0.005688	0.002976	0.004923	31894
2015	0.005679	0.002998	0.004911	33992
2016	0.005639	0.002970	0.004927	35845
2017	0.005600	0.002977	0.004914	36294
2018	0.005672	0.003011	0.004970	37072
2019	0.005627	0.002952	0.004828	39341
2020	0.005657	0.003019	0.004995	40865
2021	0.005566	0.002941	0.004949	39501
2022	0.005599	0.002977	0.004909	40112
2023	0.005605	0.003030	0.005006	39923
2024	0.005475	0.002977	0.004917	39524
2025	0.005603	0.003005	0.004897	18180

S5.6. Fig 2 C

year	mean	sd_minus	sd_plus	*N*
1963	0.0108	0.0000	0.0000	1
1970	0.0035	0.0005	0.0005	2
1972	0.0061	0.0008	0.0014	3
1975	0.0016	0.0000	0.0000	1
1976	0.0046	0.0030	0.0030	2
1977	0.0037	0.0018	0.0036	5
1980	0.0032	0.0000	0.0000	1
1982	0.0089	0.0054	0.0021	7
1983	0.0024	0.0011	0.0019	3
1984	0.0078	0.0028	0.0026	5
1985	0.0051	0.0031	0.0063	7
1986	0.0053	0.0023	0.0045	4
1987	0.0041	0.0020	0.0034	22
1988	0.0047	0.0026	0.0063	15
1989	0.0058	0.0026	0.0066	19
1990	0.0053	0.0029	0.0065	20
1991	0.0064	0.0032	0.0048	24
1992	0.0067	0.0036	0.0057	27
1993	0.0052	0.0027	0.0046	50
1994	0.0045	0.0022	0.0040	123
1995	0.0055	0.0027	0.0050	595
1996	0.0056	0.0028	0.0049	2138
1997	0.0055	0.0028	0.0049	2803
1998	0.0054	0.0027	0.0050	5233
1999	0.0055	0.0029	0.0051	10659
2000	0.0055	0.0028	0.0052	13137

2001	0.0057	0.0029	0.0051	15904
2002	0.0058	0.0029	0.0052	19289
2003	0.0058	0.0029	0.0052	22629
2004	0.0059	0.0030	0.0052	24837
2005	0.0059	0.0030	0.0052	28703
2006	0.0060	0.0030	0.0052	31983
2007	0.0061	0.0031	0.0052	33779
2008	0.0060	0.0031	0.0052	34378
2009	0.0060	0.0030	0.0052	38307
2010	0.0059	0.0030	0.0052	39630
2011	0.0060	0.0031	0.0052	42258
2012	0.0060	0.0030	0.0051	44996
2013	0.0059	0.0030	0.0052	43617
2014	0.0060	0.0031	0.0053	43587
2015	0.0059	0.0030	0.0052	45511
2016	0.0059	0.0030	0.0052	46681
2017	0.0059	0.0030	0.0052	48465
2018	0.0059	0.0030	0.0052	48666
2019	0.0059	0.0030	0.0052	49485
2020	0.0059	0.0031	0.0053	52015
2021	0.0059	0.0030	0.0053	49478
2022	0.0059	0.0031	0.0052	49350
2023	0.0059	0.0031	0.0053	51913
2024	0.0058	0.0031	0.0052	56212
2025	0.0059	0.0031	0.0052	25295

S5.7. Fig 2 D

year	mean	sd_minus	sd_plus	*N*
1963	0.024123	0.000000	0.000000	1
1970	0.007795	0.000762	0.000762	2
1972	0.010364	0.002757	0.001840	3
1975	0.003175	0.000000	0.000000	1
1976	0.012551	0.010842	0.010842	2
1977	0.003574	0.001449	0.000944	3
1980	0.007633	0.000000	0.000000	1
1982	0.022558	0.014162	0.005367	7
1983	0.004933	0.002529	0.004338	3
1984	0.017965	0.006604	0.005774	5
1985	0.012020	0.006383	0.008964	6
1986	0.013253	0.006398	0.012562	4
1987	0.008606	0.006029	0.006941	20
1988	0.011399	0.007326	0.011193	13
1989	0.012005	0.005008	0.012369	17
1990	0.011220	0.005650	0.007165	14
1991	0.013597	0.006409	0.007531	22
1992	0.015902	0.009916	0.012307	21

1993	0.014766	0.007651	0.013388	34
1994	0.010374	0.005148	0.010186	95
1995	0.013518	0.006744	0.013168	481
1996	0.013250	0.007004	0.011983	1645
1997	0.013536	0.007084	0.012129	2020
1998	0.013092	0.007069	0.012559	3455
1999	0.013521	0.007392	0.012861	6571
2000	0.013312	0.007355	0.013389	8084
2001	0.013924	0.007623	0.013196	10032
2002	0.013946	0.007630	0.013498	12248
2003	0.013975	0.007595	0.013121	14287
2004	0.014425	0.007740	0.013037	15730
2005	0.014535	0.007788	0.013118	18308
2006	0.014720	0.007860	0.013081	20613
2007	0.014839	0.007957	0.013055	21769
2008	0.014850	0.007903	0.013109	21801
2009	0.014571	0.007767	0.013040	24643
2010	0.014478	0.007747	0.012861	25105
2011	0.014723	0.007893	0.012964	27327
2012	0.014825	0.007872	0.012896	29098
2013	0.014536	0.007756	0.012945	27558
2014	0.014859	0.007902	0.013159	28245
2015	0.014740	0.007887	0.013017	29598
2016	0.014662	0.007814	0.013023	31219
2017	0.014596	0.007841	0.013109	32111
2018	0.014740	0.007912	0.013074	32829
2019	0.014714	0.007825	0.012770	34100
2020	0.014718	0.007905	0.012916	35756
2021	0.014592	0.007735	0.013076	34498
2022	0.014820	0.007906	0.012993	34881
2023	0.014716	0.007972	0.013201	36450
2024	0.014434	0.007904	0.013044	38603
2025	0.014795	0.008006	0.013118	17890

S5.8. Elemental averages

element	*Z*	R1	wR2	abs_min_density	max_density	Nr. Occur.
H	1	5.219	13.474	0.814	0.982	1220144
He	2	5.340	16.284	0.247	0.251	28
Li	3	5.831	14.990	0.606	0.813	12300
Be	4	4.942	12.473	0.474	0.589	748
B	5	5.435	14.234	2.277	2.371	94078
C	6	5.218	13.473	0.814	0.982	1223908
N	7	5.242	13.623	0.858	1.020	901965
O	8	5.287	13.720	0.835	0.992	923773
F	9	5.432	14.277	1.558	1.729	181875
Ne	10	4.544	4.707	0.501	0.382	2

Na	11	6.020	15.843	1.066	1.390	10768
Mg	12	5.790	15.307	0.503	0.679	5791
Al	13	5.518	14.337	0.575	0.779	13752
Si	14	5.141	13.036	0.712	0.937	66311
P	15	5.025	12.632	0.910	1.182	207505
S	16	5.190	13.391	1.311	1.447	255209
Cl	17	5.208	13.433	0.830	1.058	285345
Ar	18	6.973	20.029	0.591	0.844	15
K	19	5.647	14.550	0.992	1.334	12769
Ca	20	5.763	15.081	0.624	0.823	2954
Sc	21	5.970	15.572	0.614	0.798	1748
Ti	22	5.655	14.439	0.602	0.811	12323
V	23	5.635	14.885	0.724	0.952	8886
Cr	24	5.436	14.112	0.683	0.893	11936
Mn	25	5.379	13.916	0.718	0.955	19849
Fe	26	5.306	13.494	0.714	0.944	65042
Co	27	5.435	14.119	0.721	0.956	39916
Ni	28	5.366	13.890	0.697	0.935	43290
Cu	29	5.308	13.725	0.772	1.005	65173
Zn	30	5.280	14.709	0.717	0.937	28244
Ga	31	4.732	11.628	0.780	1.024	6614
Ge	32	4.850	11.980	0.867	1.145	7941
As	33	4.908	11.770	1.100	1.344	7983
Se	34	4.819	11.746	0.989	1.215	16717
Br	35	4.943	12.100	0.870	1.031	80819
Kr	36	6.014	15.365	0.594	0.726	14
Rb	37	5.703	13.371	1.105	1.431	1144
Sr	38	5.353	13.048	0.808	1.042	1188
Y	39	5.683	14.096	0.771	1.031	4608
Zr	40	4.861	12.288	0.774	1.000	8468
Nb	41	5.031	12.517	1.015	1.287	2791
Mo	42	4.872	12.115	1.032	1.301	23525
Tc	43	4.920	11.782	0.933	1.067	1060
Ru	44	4.892	12.336	0.947	1.232	38150
Rh	45	4.841	12.072	0.943	1.251	17723
Pd	46	4.869	12.529	0.961	1.227	32542
Ag	47	5.301	13.641	1.143	1.481	14308
Cd	48	4.798	12.351	0.958	1.183	7352
In	49	4.533	11.262	1.018	1.312	3401
Sn	50	4.371	10.607	0.957	1.215	16667
Sb	51	4.594	11.380	1.235	1.540	11782
Te	52	4.430	10.876	1.245	1.532	6049
I	53	4.494	10.854	1.217	1.440	42941
Xe	54	5.096	13.041	0.946	1.063	91
Cs	55	5.211	12.954	1.505	1.836	1863
Ba	56	5.023	11.953	1.112	1.461	1498
La	57	5.014	12.470	1.200	1.562	3036

Ce	58	4.753	12.197	1.224	1.622	2760
Pr	59	4.795	11.966	1.437	1.672	1404
Nd	60	4.910	12.147	1.242	1.623	3112
Pm	61	3.870	10.340	0.735	1.016	3
Sm	62	4.780	11.620	1.201	1.560	3535
Eu	63	5.021	12.624	1.277	1.656	4334
Gd	64	4.991	12.915	1.387	1.812	3650
Tb	65	4.812	12.197	1.339	1.760	3209
Dy	66	4.946	12.616	1.371	1.807	5874
Ho	67	4.534	11.287	1.419	1.772	1389
Er	68	4.665	11.695	1.379	1.773	2533
Tm	69	4.468	11.289	1.542	1.918	664
Yb	70	4.673	11.417	1.379	1.813	3992
Lu	71	4.424	10.883	1.366	1.773	1829
Hf	72	3.928	9.360	1.458	1.917	1753
Ta	73	4.167	10.142	1.645	2.123	2771
W	74	4.610	11.130	2.000	2.379	16760
Re	75	4.172	9.993	1.583	1.925	13889
Os	76	4.308	9.715	1.644	2.007	7480
Ir	77	4.199	10.027	1.564	2.072	16263
Pt	78	4.311	10.177	1.616	2.036	26670
Au	79	4.421	10.714	1.726	2.150	17749
Hg	80	4.802	10.863	1.702	1.948	5535
Tl	81	4.763	10.986	1.799	2.042	1288
Pb	82	4.419	10.104	1.658	1.925	2997
Bi	83	4.269	9.935	1.750	2.017	5019
Ra	88	4.040	9.040	0.945	1.321	2
Th	90	4.328	10.001	1.653	2.131	1714
Pa	91	4.607	8.275	1.968	3.051	8
U	92	4.472	10.693	1.698	2.146	7043
Np	93	3.939	9.097	1.593	1.867	332
Pu	94	3.826	9.109	1.618	1.921	230
Am	95	4.203	9.630	1.861	2.267	51
Cm	96	2.855	7.387	1.199	1.760	12
Bk	97	3.979	10.259	1.884	2.959	8
Cf	98	3.950	9.210	1.380	3.002	10
